# New Constituents from the Leaves of Date Palm (*Phoenix dactylifera* L.) of Saudi Origin

**DOI:** 10.3390/molecules26144192

**Published:** 2021-07-10

**Authors:** Rami K. Suleiman, Wissam Iali, Bassam El Ali, Saviour A. Umoren

**Affiliations:** 1 Interdisciplinary Research Center for Advanced Materials, King Fahd University of Petroleum & Minerals (KFUPM), Dhahran 31261, Saudi Arabia; umoren@kfupm.edu.sa; 2 Chemistry Department, King Fahd University of Petroleum & Minerals, Dhahran 31261, Saudi Arabia; wissam.iali@kfupm.edu.sa (W.I.); belali@kfupm.edu.sa (B.E.A.)

**Keywords:** characterization, date palm, isolation, phenolic, terpenes

## Abstract

The phytochemical analysis of the butanolic extract from the leaves of date palm of Saudi origin resulted in the isolation of three major constituents, oleanolic acid (**1**), vanillyl alcohol (**2**), and β-sitosterol-3-*O*-β-d-glucoside (**3**), which had not been isolated from this plant or previously reported. Together, compounds **1** and **2** account for 1.0% of the butanol extract, which represents 0.4% of the mass of the dried leaves. The isolation of other known compounds for this plant such as fatty acids, lutein, and sucrose was also achieved in this study. The characterization and identification of the isolated compounds were conducted on the basis of Fourier-transform infrared spectroscopy (FTIR), ^1^H and ^13^C nuclear magnetic resonance (NMR), liquid chromatography–mass spectrometry (LC–MS), and gas chromatography–mass spectrometry (GC–MS) analyses. The findings of the current study will definitely increase the knowledge about the contribution of the constituents of this plant to its well-known nutrition, corrosion inhibition, and antimicrobial properties.

## 1. Introduction

Research work on plants continues to provide valuable information on the compounds that can be applied in various fields, such as medicine, taxonomy, environmental pollution, corrosion protection, and nutrition. Date palm (*Phoenix dactylifera* L.) is among the best-known and extensively cultivated plants of great economic importance in Saudi Arabia. This plant belongs to the Arecaceae family and has been cultivated in the Middle East and North Africa for at least 5000 years [1]. The kingdom of Saudi Arabia has about 23 million date palms of over 400 cultivars, and its dates production is estimated to account for 17% of the total global production [2]. The different parts of this plant have been used extensively in traditional medicine to treat various disorders, such as memory disorders, inflammation, fever, loss of consciousness, diabetes, hypertension, and neurological disorders [3]. The interesting pharmacological activities of the date palm are attributed to its bioactive non-nutrient phytochemical constituents.

Earlier studies have reported that the date palm is very rich in various constituents, including fatty materials, carotenoids, polyphenols (e.g., phenolic acids, isoflavones, lignans, and flavonoids), tannins, and sterols [4,5]. The identification of some of the existing constituents in this plant was achieved based on the GC–MS (for the fatty constituents) [6], LC–MS (for the phenolic compounds) [5,7], or FTIR (for the lignans) [8] analyses. The focus of many researchers was devoted to the study of the phytochemical composition of date palm fruits and the effect of the date maturing period on its chemical constituents and functional composition [2,9,10]. However, less attention was paid by the researchers to the phytochemical composition of date palm leaves as they are of less economical, nutritional, and pharmacological importance than date fruits [11,12]. In some date palm-growing countries, palm leaves are considered to be waste materials left in agricultural lands or treated by burning [12].

Very recently, our research group reported potential applicability for the butanolic extract of date palm leaves as an inhibitive pigment for the corrosion of steel substrates in various acidic media [13,14,15]. The promising corrosion inhibition properties of the plant extract can be attributed to one or more of the individual constituents of this plant and possibly to the synergetic effect of these constituents. This encouraged us to conduct, in this study, a phytochemical analysis and identification of the chemical constituents available in the butanolic extract of the leaves of the date palm (*Phoenix dactylifera* L.) of Saudi origin.

## 2. Results and Discussion

Motivated by the interesting reported studies on the promising corrosion inhibition efficiency of various extracts of the date palm [13,14,16], we conducted in this study the phytochemical analysis of the butanolic extract of this plant. Regardless of the good number of phytochemical constituents reported in the literature for this plant [3,7,17], our research on this plant led to the isolation of constituents from this plant that are reported here for the first time. The characterization and identification of the isolated compounds in our study were as follows:

### 2.1. Fatty Acid Mixture

**Figure 1:**
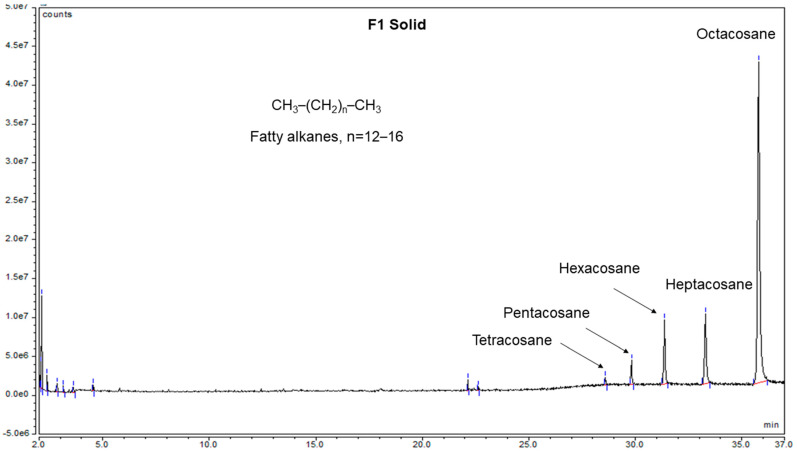
GC–MS chromatogram of the F1 solid.

The addition of methanol to F1 of the CC of the butanolic extract yielded a white solid which was analyzed by the NMR technique. The obtained ^1^H and ^13^C NMR spectra of the solid (Figures S1 and S2) indicated clearly the fatty material nature of this solid, as the observed resonances were in agreement with the literature data [18]. In order to determine the molecular mass of the components of this fatty material, we analyzed it using the GC–MS technique. The chromatogram of this sample (Figure 1) showed the presence of five major peaks identified, based on their mass spectral data, as tetracosane, pentacosane, hexacosane, heptacosane, and octacosane. The MS spectrum of octacosane (Figure S3) showed peaks corresponding to the cleavage of several –CH_2_– (*m/z* 14) linkages, which is a characteristic fragmentation pattern for fatty alkanes and acids. The identified fatty alkanes in this study were reported previously from this plant [6] and the genus *Phoenix* [1] using the GC–MS analysis of the plants’ extracts.

### 2.2. Oleanolic Acid (***1***)

**Figure 2:**
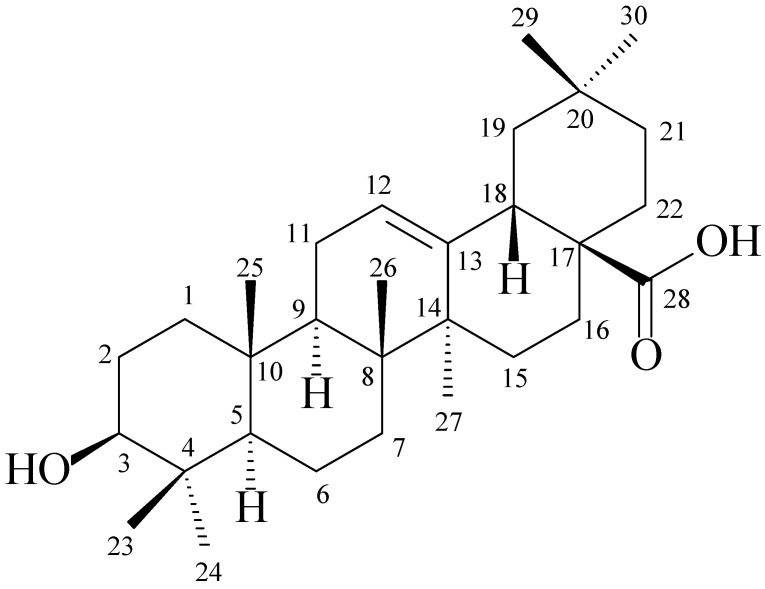
Chemical structure of **1**.

The early CC fractionations of the crude butanolic extract showed precipitation of an impure yellowish major solid, which was further purified by crystallization from methanol to yield a white pure solid identified as terpenic acid **1** (Figure 2).

The ^1^H NMR spectrum of **1** (in DMSO-*d_6_*, Figure S4) displayed singlet signals in the region δ 0.75–0.96 ppm assigned to various methyl groups in **1**. The signal of the olefinic proton at C-12 appeared at 5.23 ppm as a multiplet while the methine one at C-3 had a doublet of doublets (dd) multiplicity and a chemical shift value of 3.20 ppm. The ^13^C NMR spectrum of **1** (Figure S5) provided more structural details by showing the presence of peaks corresponding to a compound of 30 carbon atoms. The peaks at δ 179.1, 144.3, 121.9, and 77.3 ppm were characteristic signals of the terpenic acid’s chemical skeleton. The existence of ten CH_2_ groups in **1** which could be deduced from the DEPT-135 NMR spectrum of **1** (Figure S6) confirmed that our isolated compound was the oleanolic acid terpene and not its analog, the ursolic acid. The analysis of the correlation signals in the ^1^H–^1^H COSY NMR spectrum of **1** (shown in Figure S7) provided further support for its elucidated structure. The NMR data of **1** (Table 1) were in agreement with the published values [19]. It is worth mentioning here that compound **1** is known to have anti-inflammatory, antihyperlipidemic, and hepatoprotective in vivo effects. It has also been found to have antiviral and antitumor actions [20], which suggests a contributing role for compound **1** to the well-known pharmacological activities of this plant.

**Figure 3:**
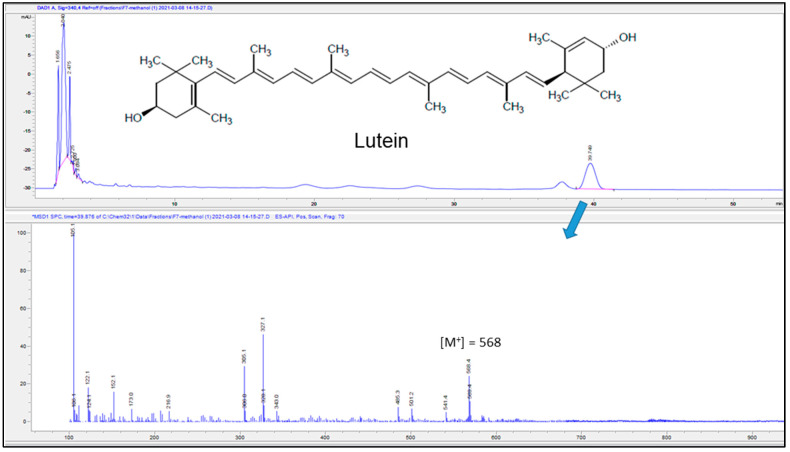
LC–MS chromatogram of lutein (**top**) along with its obtained ESI spectrum (**bottom**).

After removing all the oleanolic acid precipitate, the methanolic mother liquor of the fraction was dried to yield a yellowish oily material identified by LC–MS as the lutein carotenoid (Figure 3). The ESI spectrum of lutein showed the molecular ion peak [M^+^] at *m/z* 568 and a substantial [M+H–2H_2_O] fragment at *m/z* 541, which is a common fragmentation pattern for hydroxyl-containing carotenoids [21]. The lutein compound was reported previously from the fruits (not the leaves) of this plant [4]. Carotenoids are, in general, very sensitive to heat and light which makes their structural analysis a tedious process [21].

### 2.3. Vanillyl Alcohol (***2***)

**Figure 4:**
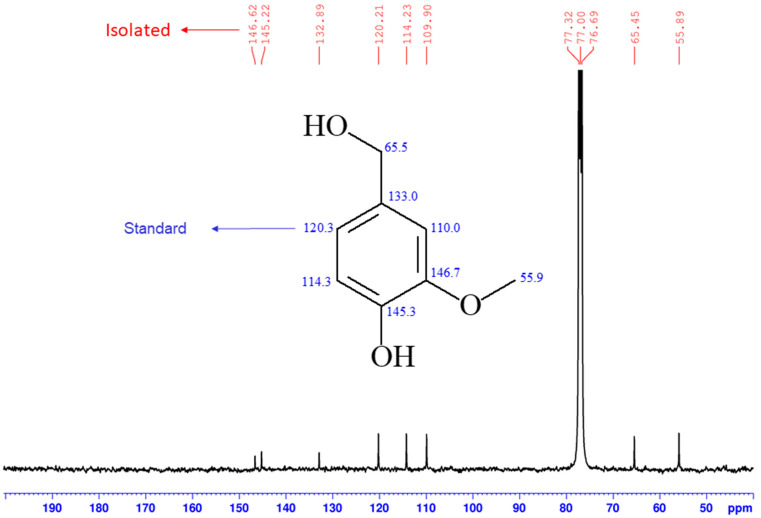
The ^13^C NMR data of the standard (blue) vs. our isolated (red) samples of **2**.

A major UV-active compound on TLC was obtained as a white solid from the CC of the butanolic extract of the studied plant, which was identified as the phenolic compound vanillyl alcohol (**2**). The ^1^H NMR spectrum of **2** (in CDCl_3_, Figure S8) showed the presence of two singlet peaks at δ 3.93 and 4.63 ppm that corresponded to the presence of OCH_3_ and OCH_2_ functionalities, respectively, in **2**. The multiplicity of the aromatic protons resonating in the region δ 6.85–6.95 ppm helped us greatly in determining the substitution pattern around the aromatic ring of **2**. The broad signal at 5.66 ppm was attributed to the Ar–OH functionality in **2**. The ^13^C NMR data of **2** were compared with the vanillyl alcohol standard and a full agreement of the data of the two samples was obtained (Figure 4). The isolation of **2** from the date palm is reported here for the first time.

It is worth mentioning here that our study found compound **2** to be a constituent of the highest quantity in the butanolic extract of the date palm. This result and the fact that many other phenolic compounds such as gallic acid, protocatechuic acid, caffeic acid, and ferulic acid have also been isolated previously from this plant [3] might explain the high lignin content identified and reported previously in the pruned *Phoenix* date palm woods [8]. Phenolic compounds are considered to be the precursors of the formation of lignin polymers in plants via a natural polymerization process that links the precursors with ether (C–O–C) linkages and with the carbon–carbon (C–C) linkage [8].

### 2.4. β-Sitosterol-3-O-β-d-Glucoside (***3***)

**Figure 5:**
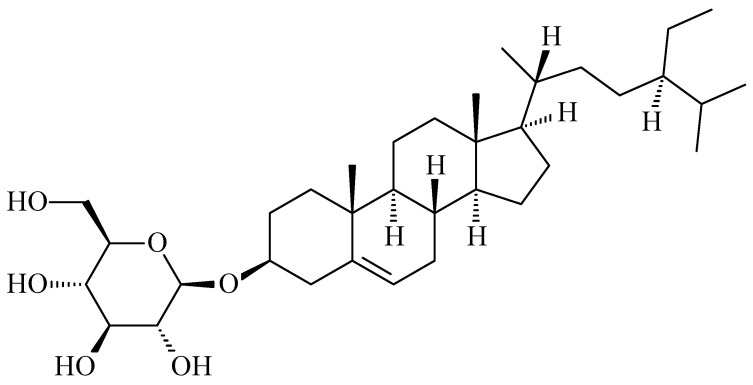
Chemical structure of **3**.

The addition of methanol to F4 of the CC of the butanolic extract resulted in a pure white solid identified by ^1^H and ^13^C NMR analyses as glycosidic sterol **3** (Figure 5).

The ^1^H NMR spectrum of **3** in DMSO-*d_6_* (Figure S9) showed peaks in the range δ 0.67−0.94 ppm that corresponded to the methyl groups in **3**. The protons of the glucose moiety resonated in the range δ 2.88–4.44 ppm. The anomeric proton of the glucose–sterol linkage was observed as a doublet (*J* = 8.87 Hz) at δ 4.20 ppm indicating a configuration for this linkage. The ^13^C NMR spectrum of compound **3** (Figure S10) confirmed the presence of 35 signals; six were for the sugar moiety and 29 were attributed to the aglycone moiety. The peak of the anomeric carbon resonated at δ 101.2 ppm while the signals of the olefinic carbons of compound **3** appeared at δ 121.7 and 140.9 ppm. The NMR spectral data of compound **3** were in full agreement with those reported in the literature [22]. This is the first report of compound **3** from the date palm.

Fraction F5 yielded, upon the addition of chloroform, a white crystalline material identified by 1D and 2D NMR techniques (Figures S11–S15) as the naturally produced sucrose sugar. The NMR data of the isolated sucrose were in full agreement with those reported in the literature [23]. Although the fruits of the date palm are rich in various types of natural sugars including sucrose, it is also interesting to report that such materials can be obtained from the leaves [24].

It is worthy to note that the evaluation of the corrosion inhibition properties of the isolated compounds in this study is ongoing and the results will be published later.

## 3. Materials and Methods

### 3.1. Chemicals

All the chemicals used in the extraction and column chromatography (CC) procedures were of analytical grade, obtained from Sigma-Aldrich (St. Louis, MO, USA), and used as received. HPLC-grade solvents and a 0.45 µm-filtered distilled water were used in the preparation of the mobile phase for the LC–MS analysis. 

### 3.2. Plant Material

Date palm leaves were collected from the city of Dhahran, Saudi Arabia, in May 2020 and kept to dry at room temperature and open to the air for one week. The dried leaves were powdered prior to extraction.

### 3.3. Butanol Extraction and Isolation

The fine powder of the dried date palm leaves (140 g) was subjected to butanolic extraction in the 1:3 (powder/solvent) ratio using a one-liter beaker under continuous stirring at 200 rpm and 25 °C for 48 h. The crude butanolic extract was filtered with double Whatman filter papers No. 1 and concentrated by evaporation under reduced pressure over a water bath at 60 °C to afford 60 g of greenish extract. This extract was purified by CC using ethyl acetate as an eluent and increasing polarity with methanol to give seven fractions (F1–F7). The addition of methanol to F1 yielded 51.6 mg of a pure white solid which was identified by NMR and GC–MS as a mixture of fatty alkanes (tetracosane, pentacosane, hexacosane, heptacosane, and octacosane). Fractions F2 and F4 were further purified using CC whereas F3 was purified by washing with methanol. Fraction F2 (9.5 g) was further purified by CC using hexane as an eluent and increasing polarity with ethyl acetate then methanol to provide five fractions according to their TLC behavior. Subfraction F2-2 showed a UV-inactive compound which was further purified by washing with methanol to yield 217.7 mg of a white solid identified as oleanolic acid (**1**). F2-4 showed a dark yellow spot on TLC which was identified by the LC–MS analysis as the carotenoid lutein. The addition of methanol to F3 and subfraction F2-5 afforded 373.7 mg of a UV-active compound identified as vanillyl alcohol (**2**). F4 showed a major UV-inactive compound (a pink-colored spot on TLC after spraying with an anisaldehyde TLC visualization reagent), which was further purified by washing with methanol to afford 9.2 mg of β-sitosterol-3-*O*-β-d-glucoside (**3**). Finally, the addition of methanol to F5 yielded 35.0 mg of a white material identified as the natural sugar sucrose. Fractions F6 and F7 did not show any major spots, therefore, they were not investigated any further.

### 3.4. NMR Spectroscopy

All the ^1^H and ^13^C NMR spectra were recorded on a Bruker Avance 400 MHz spectrometer (Bruker, Billerica, MA, USA) at 400 and 100 MHz, respectively. CDCl_3_ or DMSO-*d_6_* were used to dissolve the samples and the chemical shifts were recorded in parts per million (δ) with tetramethylsilane (TMS) as an internal reference. The 2D NMR homonuclear ^1^H/^1^H correlation spectroscopy (TOCSY, COSY) and heteronuclear ^13^C/^1^H correlation experiments (HMBC, HETCOR) were conducted using the standard Bruker pulse sequence. More details on the acquisition parameters of the NMR experiments conducted in this study are listed in Table S1 in the Supporting Information.

### 3.5. GC–MS Parameters

The GC–MS analysis of F3 was carried out using an ISQ™ 7000 Single Quadrupole GC–MS System (ThermoFisher Scientific, Milan, Italy). A TraceGOLD TG-5MS GC capillary column (5% phenyl and 95% dimethylpolysiloxane) with 30 m × 0.25 mm × 0.25 μm dimensions was equipped with the instrument. The temperature in the column was programmed at 50 °C, ramped up at 10 °C min^−1^ to 150 °C, held isothermal for 2 min, ramped up at 10 °C min^−1^ to 250 °C, and held then for 10 min. Highly pure helium was used as a carrier gas at the flow rate of 0.85 mL min^−1^. The injector temperature was 250 °C; the volume of samples injected was 1 μL in the split mode (50:1). Electron ionization was effected by the electron impact (EI) with the ionization energy of 70 eV. The interface temperature was set at 250 °C and the ionization source was 200 °C. The analyzer is quadrupole-type (250 °C). The mass spectrum was recorded using the mass detector scan mode (range, 50–800 amu). The identification of chemical compounds was performed by comparison of their mass spectra with those registered in the NIST2017 database.

### 3.6. LC–MS Parameters

Some of the fractions obtained from the CC of the butanolic extracts were subjected to the LC–MS analysis, which was performed on Agilent LCMS-6120B (Agilent, Santa Clara, CA, USA) coupled with a diode-array detector (DAD) using a Poroshell 120 EC-C18 (4.6 × 150 mm, 4 µm) column. The temperature of the column oven was kept at 25 °C. Isocratic elution was employed for the analysis, with a mobile phase consisting of 0.1% formic acid in water (solution A, 80%) and methanol (solution B, 20%), with the post-time of 5 min before the next injection. The flow rate of the mobile phase was 1.0 mL/min, and the injection volume was 20 µL of the sample. 

## 4. Conclusions

In conclusion, the isolation of six constituents from the leaves of the *Phoenix dactylifera* L. plant of Saudi origin was successfully achieved in this study. Regardless of the extensive phytochemical analyses conducted on this plant and reported previously in the literature, our research work led here to the isolation of three compounds, triterpene, phenolic, and sterol–glycoside, from this plant for the first time. The isolated vanillyl alcohol component in this study suggests its incorporation in the biosynthesis of lignin polymers produced by this plant. This study contributes to the validity of the significant role of the type of the extracting solvent on the nature of the isolated constituents from plants. Moreover, the phytochemicals isolated from the highly abundant date palm leaves and their potential applications can be economically of considerable importance to the date industry. Further studies on evaluating the corrosion inhibition properties of the compounds isolated in this work are ongoing. 

## Figures and Tables

**Table 1:** Experimental ^13^C and ^1^H NMR data for compound **1** and data from the literature.

Position	Experimental		Literature [19]	
	^13^C	^1^H	^13^C	^1^H
C-1	38.5	1.62 (m)	38.5	1.63 (m)
C-2	28.6	1.59 (m)	28.1	1.59 (m)
C-3	77.3	3.20 (dd; *J* = 4.0; 8.0 Hz)	79.1	3.20 (dd; *J* = 4.5; 10.0 Hz)
C-4	38.5	-	38.8	
C-5	55.2	0.66 (m)	55.2	0.71 (m)
C-6	18.5	1.54 (m)	18.2	1.54 (m)
C-7	32.9	1.47 (m)	32.9	1.48 (m)
C-8	38.8	-	39.4	
C-9	45.9	1.54 (m)	47.5	1.54 (m)
C-10	37.1	-	37.0	
C-11	23.4	0.93 (m)	23.5	0.94 (m)
C-12	122.0	5.23 (m)	125.8	5.23 (d; *J* = 22.5 Hz)
C-13	144.3	-	137.9	
C-14	41.8	-	41.9	
C-15	27.7	1.61 (m)	27.9	1.61 (m)
C-16	23.8	0.96 (m)	24.1	0.96 (m)
C-17	47.5	-	47.8	
C-18	41.2	-	41.9	
C-19	46.1	-	47.5	
C-20	30.9	-	30.6	
C-21	38.8	1.62 (m)	38.8	1.63 (m)
C-22	33.8	1.31 (m)	36.9	1.31 (m)
C-23	27.6	0.99 (s)	28.1	0.96 (s)
C-24	16.5	0.75 (s)	16.9	0.75 (s)
C-25	15.6	0.91 (s)	15.4	0.91 (s)
C-26	17.2	0.76 (s)	17.0	0.76 (s)
C-27	27.4	1.13 (s)	27.2	1.06 (s)
C-28	179.0	-	181.2	
C-29	33.3	0.90 (s)	36.7	0.84 (s)
C-30	23.3	0.93 (s)	23.2	0.93 (s)

## Data Availability

The data presented in this study are available upon request from the corresponding author.

## Supplementary Materials

The following are available online. Table S1: Selected parameters of the conducted NMR experiments, Figures S1–S15: ^1^H NMR spectrum, ^13^C NMR spectrum, ^1^H-^13^C HMQC NMR spectrum.

## Sample Availability

Samples of the compounds are not available from the authors.

## References

[1] Hifnawy M., Mahrous A., Ashour R. (2016). Phytochemical investigation of Phoenix canariensis Hort. ex Chabaud leaves and pollen grains and spathe volatile analysis. J. Appl. Pharm. Sci..

[2] Ali H., Al-Khalifa A.R., Farouk A., Shaheen M. (2018). Effect of Maturation Stages on Flavor Profile and Antioxidant Activity of Date Palm Fruits (*Phoenix dactylifera*) Grown in Saudi Arabia. Int. J. Pharmacol..

[3] Vyawahare N., Pujari R., Khsirsagar A., Ingawale D., Patil M., Kagathara V. (2009). Phoenix dactylifera: An update of its indegenous uses, phytochemistry and pharmacology. Internet J. Pharmacol..

[4] Al-Alawi R.A., Al-Mashiqri J.H., Al-Nadabi J.S.M., Al-Shihi B.I., Baqi Y. (2017). Date Palm Tree (*Phoenix dactylifera* L.): Natural Products and Therapeutic Options. Front. Plant Sci..

[5] Alshwyeh H.A. (2020). Phenolic profiling and antibacterial potential of Saudi Arabian native date palm (*Phoenix dactylifera*) cultivars. Int. J. Food Prop..

[6] Arif Y., Lombarkia N. (2018). Variability Influence of the Volatile Compounds of Three Algerian Date Cultivars (*Phoenix dactylifera* L.) on Infestation Rates of the Date Moth [(Ectomyelois ceratoniae Zell. (Lepidoptera: Pyralidae)]. Cercet. Agron. Mold..

[7] Abu-Reidah I.M., Gil-Izquierdo Á., Medina S., Ferreres F. (2017). Phenolic composition profiling of different edible parts and by-products of date palm (*Phoenix dactylifera* L.) by using HPLC-DAD-ESI/MSn. Food Res. Int..

[8] Abdullah H.M., Abdul Latif M.H., Attiya H.G. (2013). Characterization and determination of lignin in different types of Iraqi phoenix date palm pruning woods. Int. J. Biol. Macromol..

[9] Alturki S., Shahba M., Stushnoff C. (2010). Diversity of antioxidant properties and phenolic content of date palm (Phoenix dactylifera L.) fruits as affected by cultivar and location. J. Food Agric. Environ..

[10] Al-Farsi M., Morris A., Baron M. (2007). Functional Properties of Omani Dates (*Phoenix dactylifera* L.). Acta Hortic..

[11] Nasser R., Salem M., Hiziroglu S., Al-Mefarrej H., Mohareb A., Alam M., Aref I. (2016). Chemical Analysis of Different Parts of Date Palm (*Phoenix dactylifera* L.) Using Ultimate, Proximate and Thermo-Gravimetric Techniques for Energy Production. Energies.

[12] Jonoobi M., Shafie M., Shirmohammadli Y., Ashori A., Zarea-Hosseinabadi H., Mekonnen T. (2019). A Review on Date Palm Tree: Properties, Characterization and Its Potential Applications. J. Renew. Mater..

[13] Umoren S.A., Solomon M.M., Obot I.B., Suleiman R.K. (2018). Comparative studies on the corrosion inhibition efficacy of ethanolic extracts of date palm leaves and seeds on carbon steel corrosion in 15% HCl solution. J. Adhes. Sci. Technol..

[14] Umoren S.A., Solomon M.M., Obot I.B., Suleiman R.K. (2021). Date palm leaves extract as a green and sustainable corrosion inhibitor for low carbon steel in 15 wt.% HCl solution: The role of extraction solvent on inhibition effect. Environ. Sci. Pollut. Res..

[15] Umoren S.A., Solomon M.M., Obot I.B., Suleiman R.K. (2021). Effect of Intensifier Additives on the Performance of Butanolic Extract of Date Palm Leaves against the Corrosion of API 5L X60 Carbon Steel in 15 wt.% HCl Solution. Sustainability.

[16] Umoren S.A., Gasem Z.M., Obot I.B. (2013). Natural Products for Material Protection: Inhibition of Mild Steel Corrosion by Date Palm Seed Extracts in Acidic Media. Ind. Eng. Chem. Res..

[17] Ghnimi S., Umer S., Karim A., Kamal-Eldin A. (2017). Date fruit (*Phoenix dactylifera* L.): An underutilized food seeking industrial valorization. NFS J..

[18] Fadzil S.R., Yap A.-C., Choo Y.-M. (2017). A New Cyclopentylidene and Other Chemical Constituents from Malaysian *Crotalaria pallida*. Sains Malays..

[19] Samarakoon S.R., Ediriweera M.K., Wijayabandara L., Fernando N., Tharmarajah L., Tennekoon K.H., Piyathilaka P., Adhikari A. (2018). Isolation of cytotoxic triterpenes from the mangrove plant, *Scyphiphora hydrophyllacea* C.F.Gaertn (Rubiaceae). Trop. J. Pharm. Res..

[20] Liby K.T., Yore M.M., Sporn M.B. (2007). Triterpenoids and rexinoids as multifunctional agents for the prevention and treatment of cancer. Nat. Rev. Cancer.

[21] Rezanka T., Olsovska J., Sobotka M., Sigler K. (2009). The Use of APCI-MS with HPLC and Other Separation Techniques for Identification of Carotenoids and Related Compounds. Curr. Anal. Chem..

[22] Peshin T., Kar H. (2017). Isolation and Characterization of β-Sitosterol-3-O-β-D-glucoside from the Extract of the Flowers of Viola odorata. Br. J. Pharm. Res..

[23] Hernández-García E., García A., Avalos-Alanís F.G., Rivas-Galindo V.M., Delgadillo-Puga C., del Rayo Camacho-Corona M. (2019). Nuclear magnetic resonance spectroscopy data of isolated compounds from *Acacia farnesiana* (L) Willd fruits and two esterified derivatives. Data Brief.

[24] Lockwood R. (2013). *Dates: Production, Processing, Food, and Medicinal Values (Medicinal and Aromatic Plants—Industrial Profiles)*; Manickavasagan, A., Essa, M. M., Sukumar, E., Eds.; CRC Press: Boca Raton, FL, USA, 2012; pp. xviii + 415, ISBN 978-1-4398-4945-3. Exp. Agric..

