# Supplementary material for: New Constituents from the Leaves of Date Palm (Phoenix dactylifera L.) of Saudi Origin
Source: Molecules. 2021 Jul 10;26(14):4192. doi: 10.3390/molecules26144192 (PMC8306910; doi:10.3390/molecules26144192)
Supplement: Supplementary file 1 [file molecules-26-04192-s001.zip › molecules-1283480-supplementary.pdf]

Supplementary data

New Constituents from the Leaves of Date Palm (Phoenix Dactylifera L.) of Saudi Origin

Rami K. Suleiman[a]*, Wissam Iali[b], Bassam El Ali[b], Saviour A. Umoren[a]

[a] Interdisciplinary Research Center for Advanced Materials, King Fahd University of Petroleum & Minerals (KFUPM), Dhahran 31261, Saudi Arabia

[b] Chemistry Department, King Fahd University of Petroleum & Minerals, Dhahran 31261, Saudi Arabia

Table S1: Selected parameters of the conducted NMR experiments.

**Parameter**	**NMR Experiment**			
	**^{1}H**	**^{13}C**	**^{1}H-^{13}C HMBC**	**^{1}H-^{1}H COSY**
Pulse Width (PW, in us)	14	9.6	14 for H and 9.6 for C	13.88
Level Power (PL1, in W)	7.7	36.45	7.7 for H and 36.45 for C	7.7
Relaxation Delay (D1, in s)	3	10	1.5	1.5
Acquisition Time (AQ, in s)	4	1.4	0.13	0.13
Receive gain (RG)	51	203	203	203
Number of scans (NS)	16	16000	16	2
Temperature (in K)	298	298	298	298

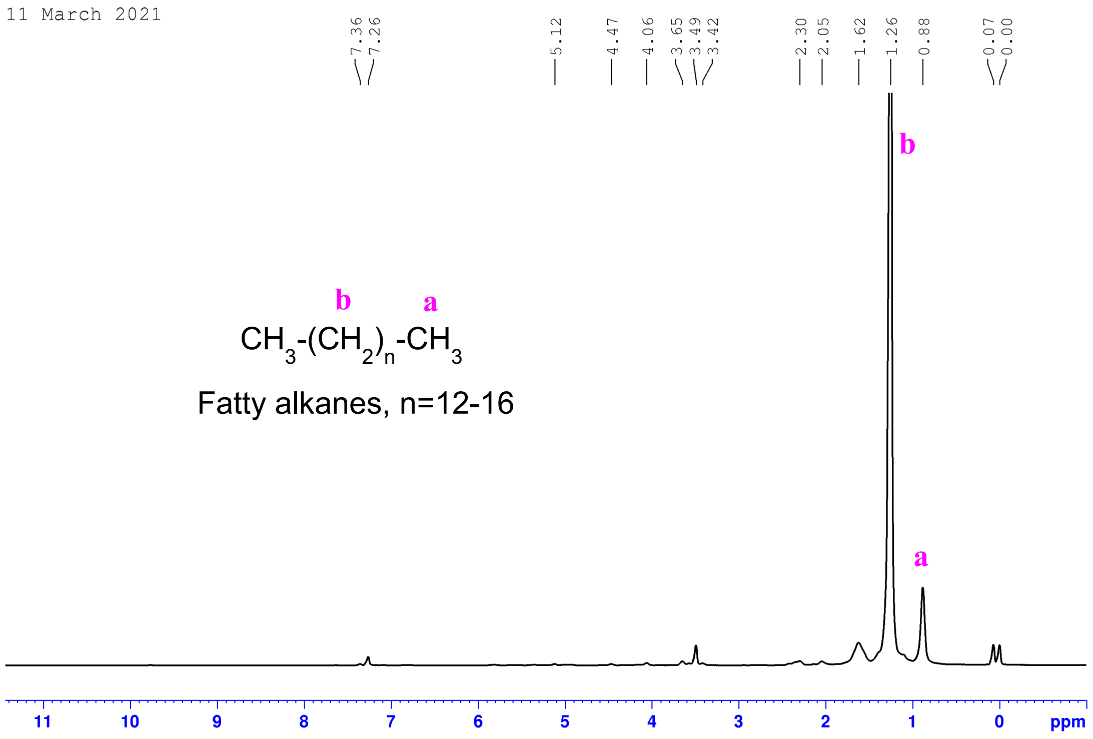

Figure S1. ^{1}H NMR spectrum of the solid of F1.

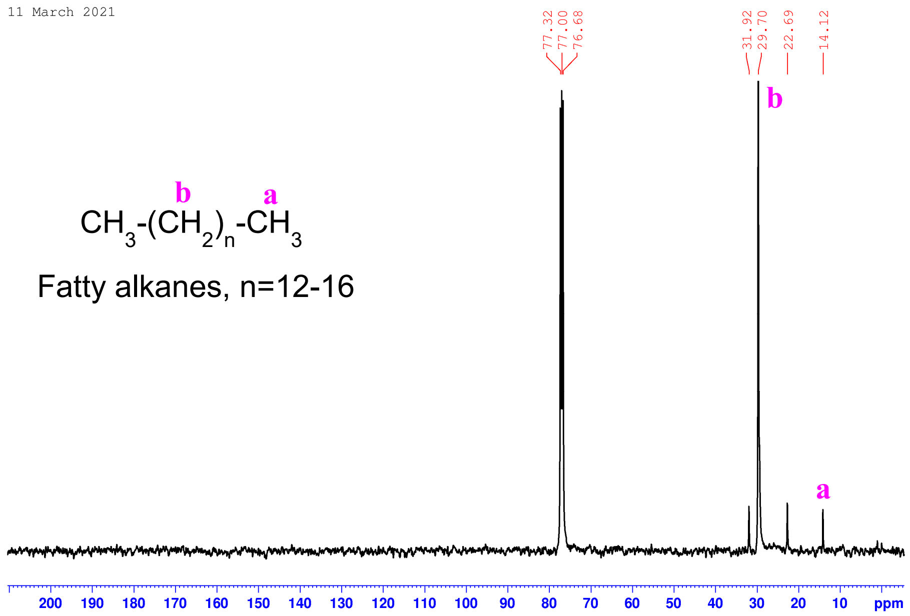

Figure S2. ^{13}C NMR spectrum of the solid of F1.

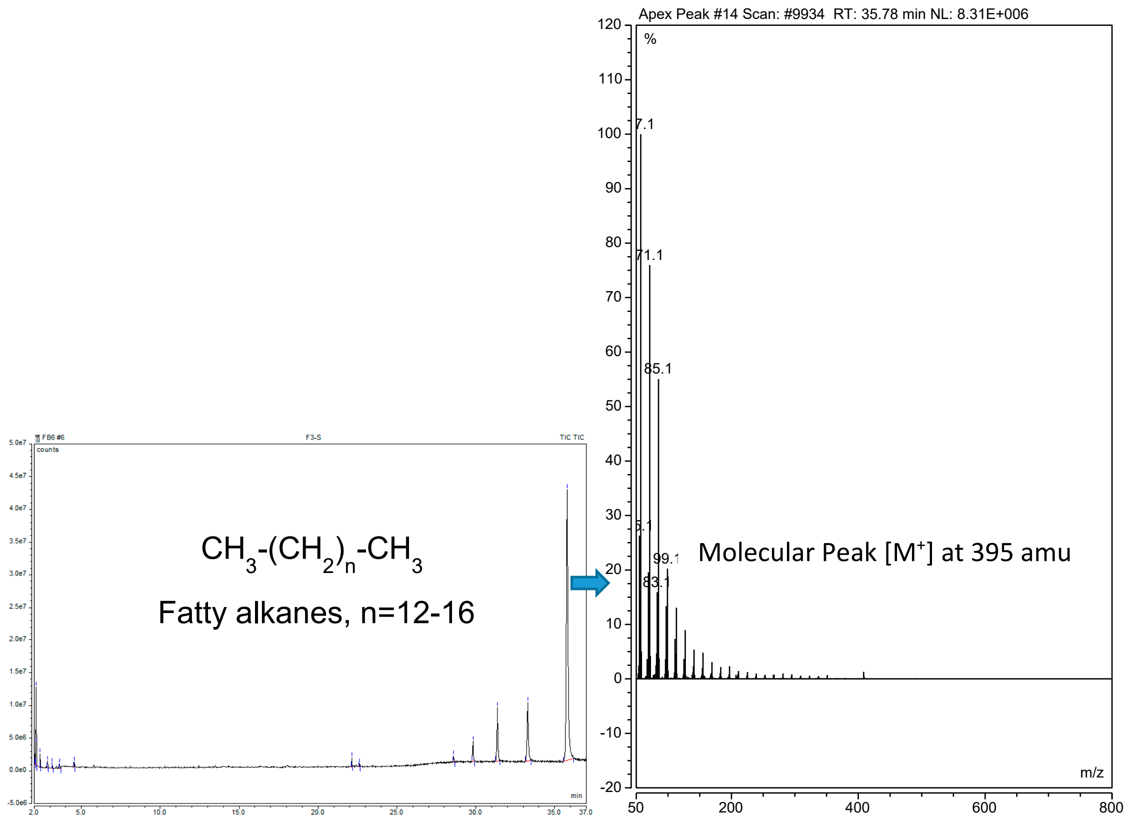

Figure S3. GC-MS Chromatogram of the solid of F1 (left) and the MS data of the highest-area peak (right).

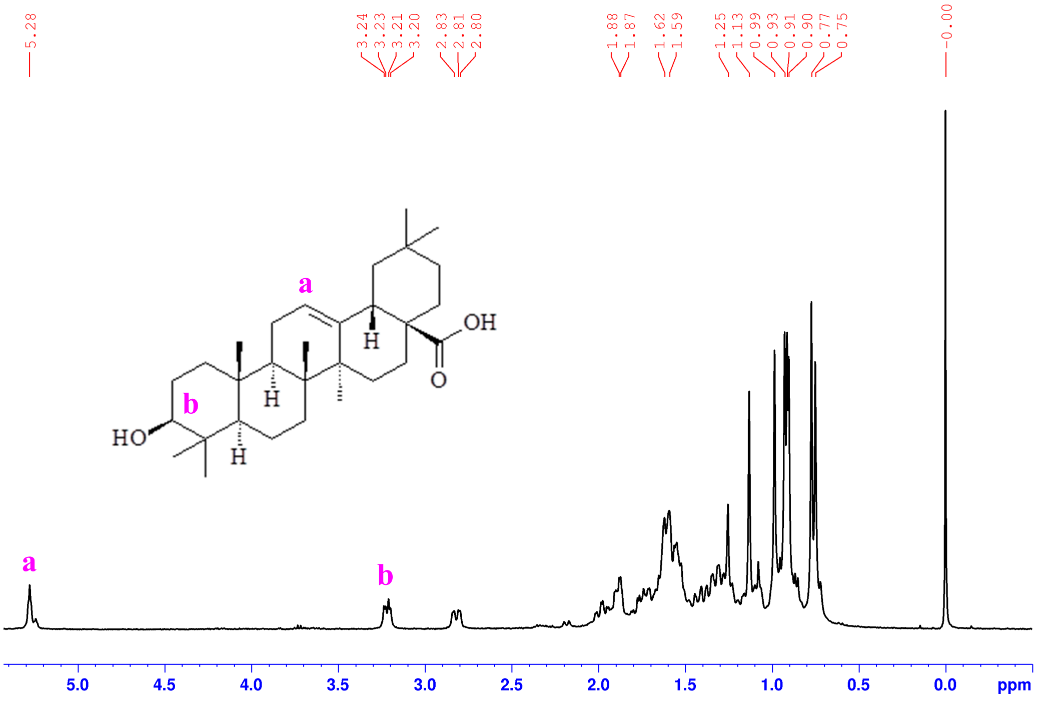

Figure S4. ^{1}H NMR spectrum of oleanolic acid.

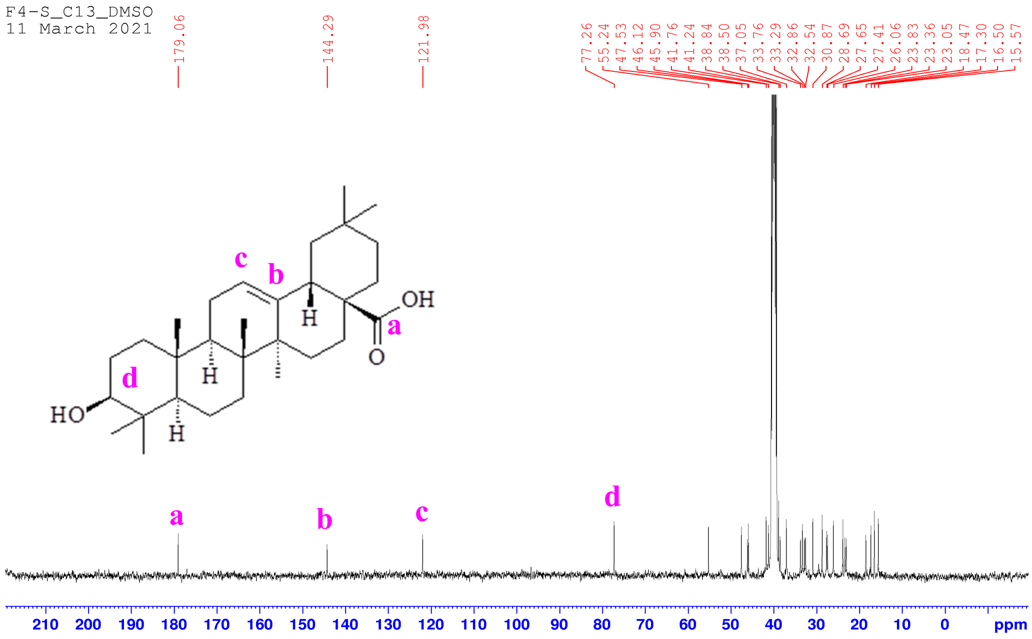

Figure S5. ^{13}C NMR spectrum of oleanolic acid.

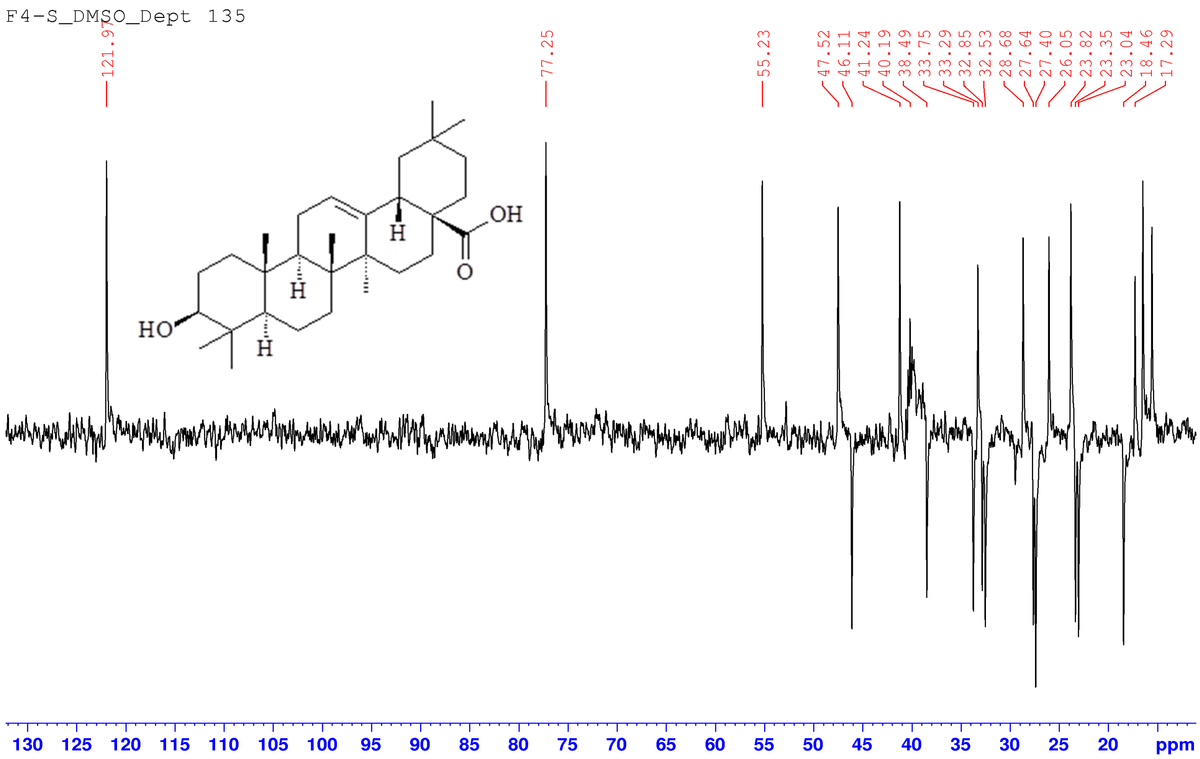

Figure S6**.** DEPT-135 NMR spectrum of oleanolic acid.

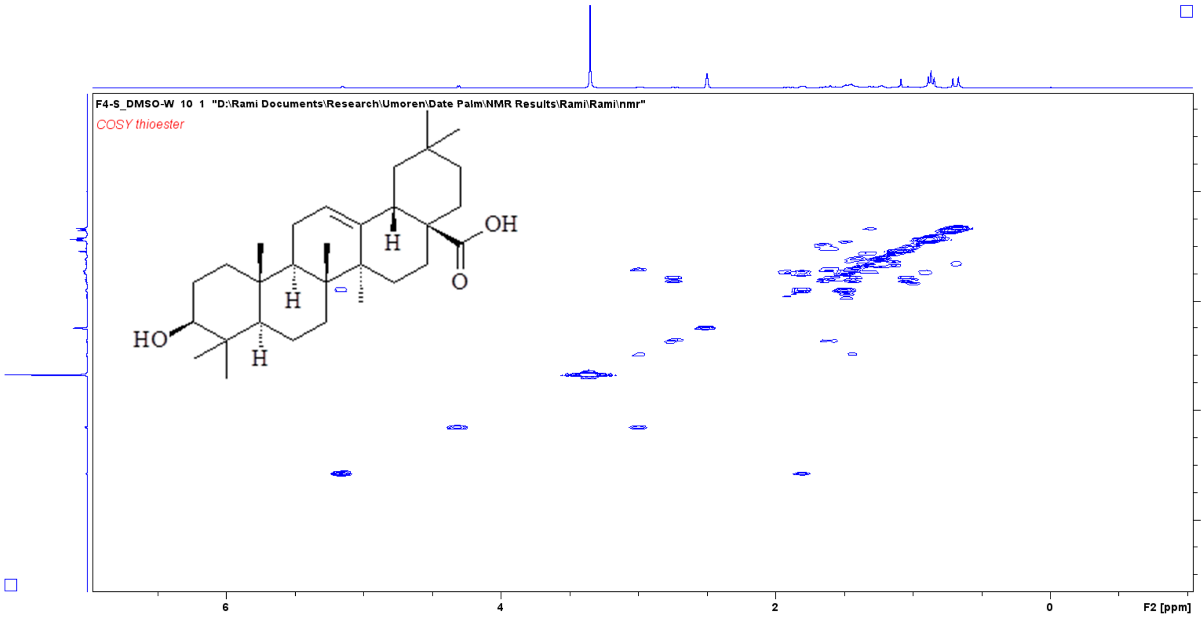

Figure S7**.** ^{1}H-^{1}H COSY NMR spectrum of oleanolic acid.

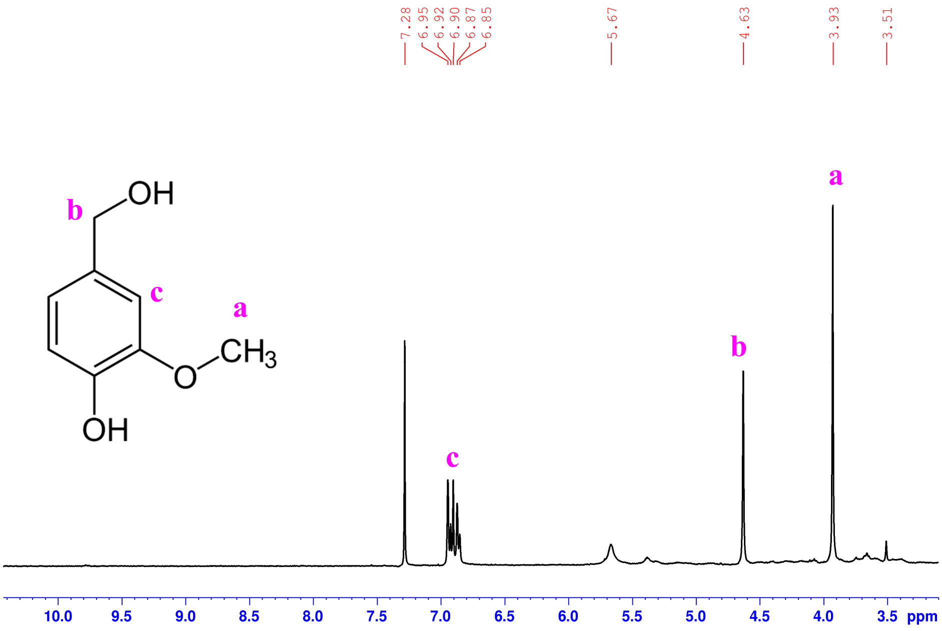

Figure S8. ^{1}H NMR spectrum of vanillyl alcohol.

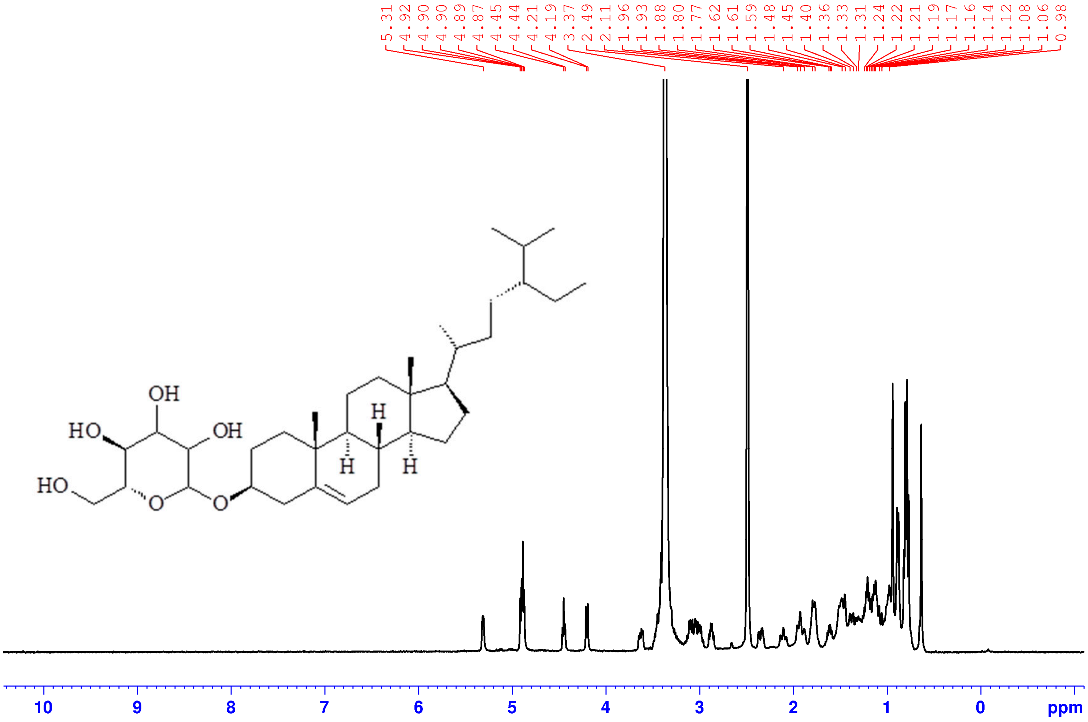

Figure S9. ^{1}H NMR spectrum of β-Sitosterol-3-O-β-D-glucoside.

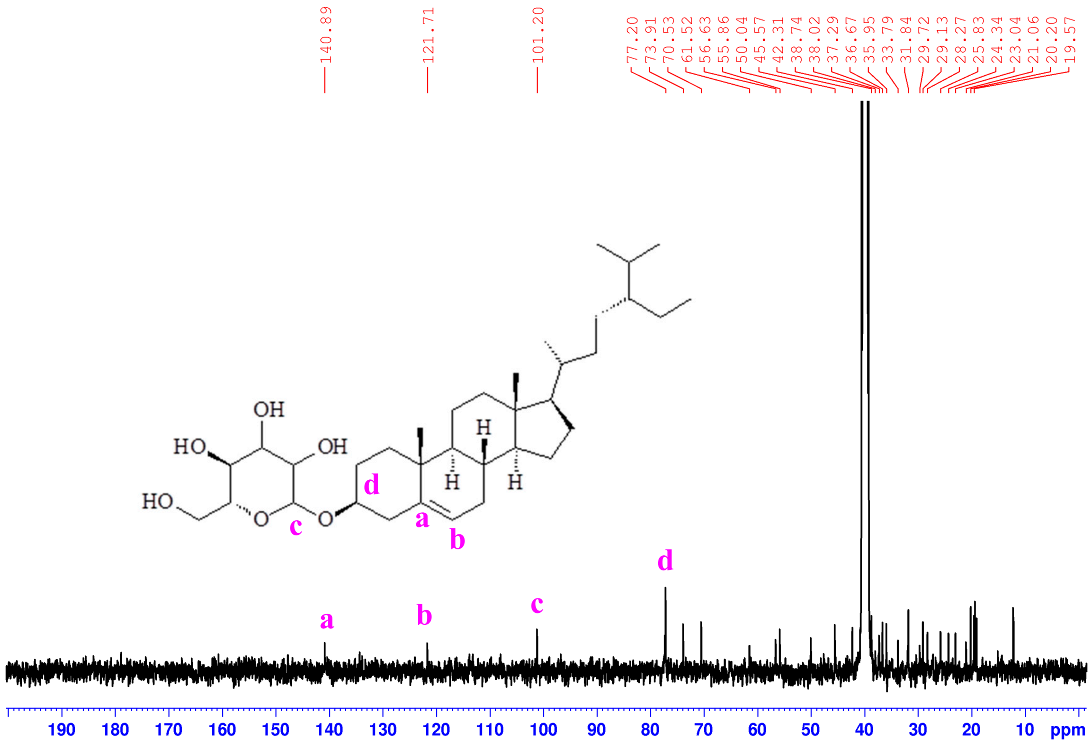

Figure S10. ^{13}C NMR spectrum of β-Sitosterol-3-O-β-D-glucoside.

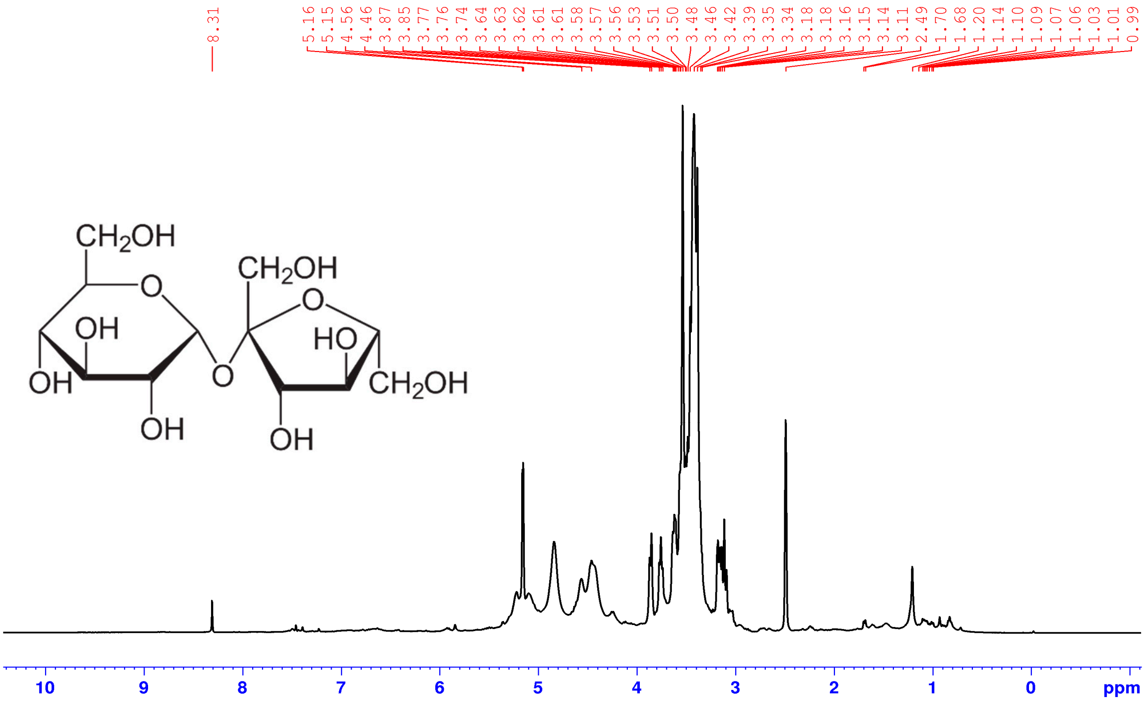

Figure S11. ^{1}H NMR spectrum of Sucrose.

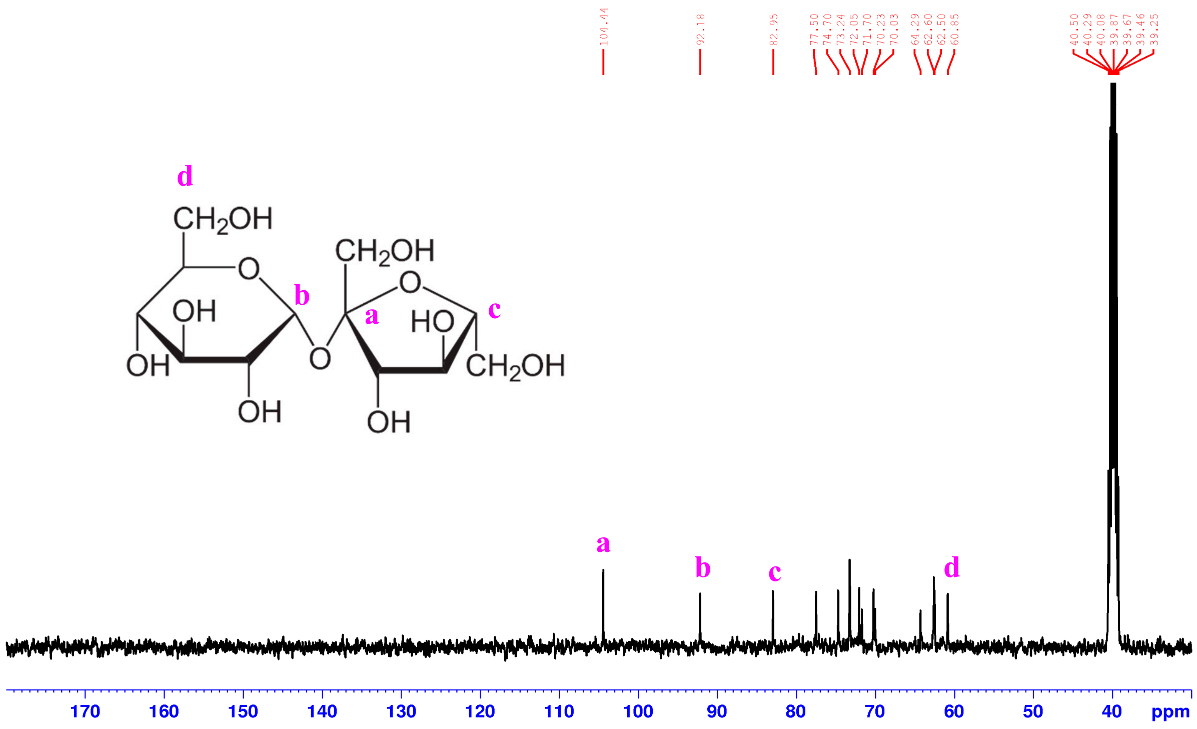

Figure S12. ^{13}C NMR spectrum of Sucrose.

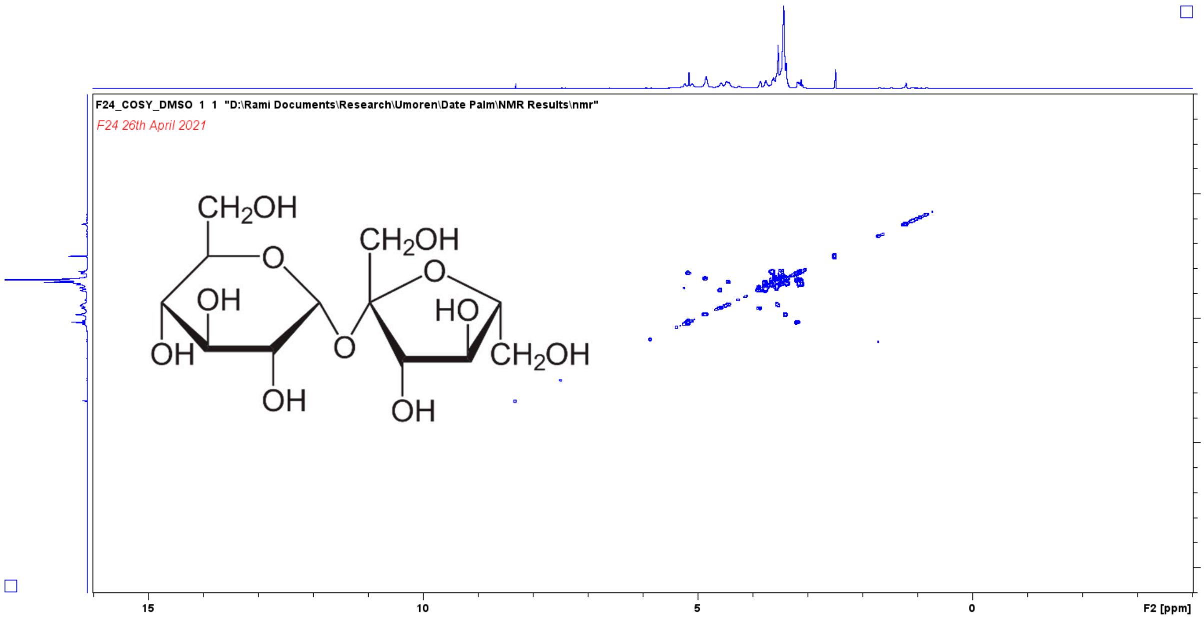

Figure S13. ^{1}H-^{1}H COSY NMR spectrum of Sucrose.

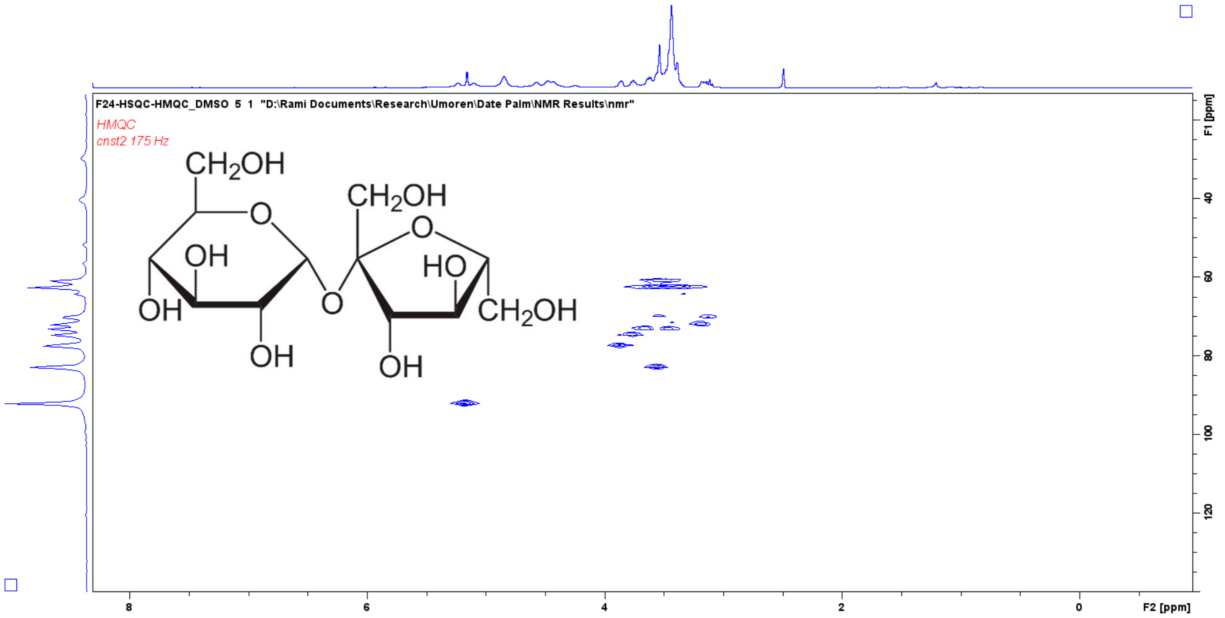

Figure S14. ^{1}H-^{13}C HMQC NMR spectrum of Sucrose.

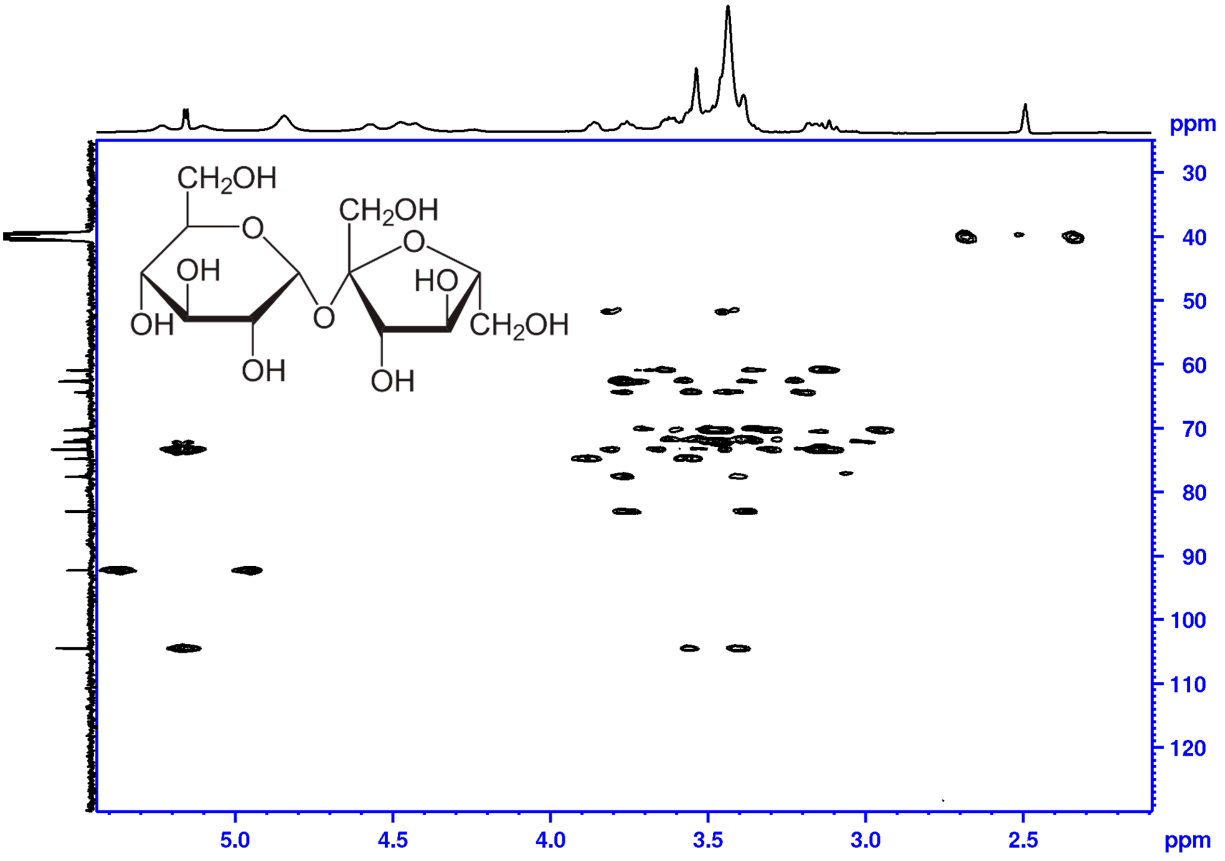

Figure S15. ^{1}H-^{13}C HMBC NMR spectrum of Sucrose.
